# EARLY DIAGNOSIS OF ALZHEIMER’S DISEASE USING A SIMPLE AND SENSITIVE NANOBIOSENSOR

**DOI:** 10.1093/geroni/igae098.3655

**Published:** 2024-12-31

**Authors:** Xiaohu Xia

**Affiliations:** University of Central Florida, Orlando, Florida, United States

## Abstract

Existing diagnostic methods for Alzheimer’s disease (AD), such as PET/MRI imaging and cerebrospinal fluid tests, are invasive and/or costly. Recent studies have demonstrated that exosomes (nanoscale extracellular vesicles carrying specific biomarkers that can traverse blood-brain barrier) are ideal sources of AD biomarkers, which can be conveniently collected from peripheral body fluids like blood and saliva. Detection of AD exosomal biomarkers opens a new avenue for non-invasive diagnosis of AD. However, the concentrations of AD exosomal biomarkers in body fluids are extremely low. Therefore, highly sensitive analytical techniques such as mass spectrometry are required to measure the exosomal biomarkers accurately. Unfortunately, these sensitive techniques come with high costs and can be cumbersome, limiting their widespread use in clinical settings. In this research, we report a simple and low-cost nano-biosensor for ultrasensitive detection of AD exosomal biomarkers. The nano-biosensor relies on metal nanoparticles as labels or signal transducers. After conjugation of the metal nanoparticles with bioreceptors specific to AD exosomal biomarkers, the nanoparticle-bioreceptor conjugates can specifically catalyze the formation of colorimetric signal that can be conveniently quantified by an inexpensive spectrophotometer. The nano-biosensor is expected to be able to detect AD exosomal biomarkers from blood and/or saliva samples, representing a minimally invasive and accessible technology for early diagnosis of AD.

